# Dietary diversity and its determinants among children aged 6–23 months in Ethiopia: evidence from the 2016 Demographic and Health Survey

**DOI:** 10.1017/jns.2022.87

**Published:** 2022-10-06

**Authors:** Binyam Girma Sisay, Tsion Afework, Beshada Rago Jima, Nardos Wondafrash Gebru, Addisalem Zebene, Hamid Y. Hassen

**Affiliations:** 1 Department of Nutrition and Dietetics, School of Public Health, Addis Ababa University, Addis Ababa, Ethiopia; 2 Department of Preventive Medicine, School of Public Health, Addis Ababa University, Addis Ababa, Ethiopia; 3 Department of Primary and Interdisciplinary Care, Faculty of Medicine and Health Sciences, University of Antwerp, Antwerp, Belgium

**Keywords:** Children and malnutrition, Determinants, Dietary diversity

## Abstract

Dietary diversity in children may be influenced not only by individual circumstances but also by the features of the community in which they live. Our study aimed to assess community and individual-level determinants of minimum dietary diversity among children aged 6–23 months in Ethiopia. We included 2960 children aged 6–23 months from the recent Ethiopia Demographic and Health Survey. A minimum dietary diversity was defined as the consumption of at least five food groups out of the eight reference food groups within 24 h by children aged 6–23 months. Multilevel logistic regression was used to investigate the drivers of minimum dietary diversity in Ethiopian children aged 6–23 months. About 12⋅5 % of children met the bare minimum of dietary diversification. Age of the child (9–11 months AOR, 3⋅3 (95 % CI 1⋅8, 5⋅6), 12–17 months AOR, 4⋅0 (95 % CI 2⋅4, 6⋅7), 18–23 months AOR, 3⋅5 (95 % CI 2⋅0, 5⋅8)), caregiver listening radio at least once a week AOR, 1⋅6 (95 % CI 1⋅1, 2⋅4) and wealth quantiles (Second AOR, 1⋅8 (95 % CI 1⋅1, 3⋅1), Fourth AOR, 2⋅9 (95 % CI 1⋅6, 5⋅2) and Highest AOR, 2⋅2 (95 % CI 1⋅1, 4⋅2)) were individual characteristics associated with dietary diversity. Place of residence was the only community-level characteristic associated with children's dietary diversity (Rural AOR, 0⋅4 (95 % CI 0⋅2, 0⋅6)). The minimum dietary diversity among Ethiopian children is suboptimal. Nutrition programmes aimed at enhancing dietary diversity should be strengthened in this population, particularly for those from poor families and residing in rural areas.

## Introduction

Globally, childhood malnutrition poses a serious public health challenge^(1)^. Undernutrition is responsible for approximately 45 % of deaths among children under the age of five, particularly in low- and middle-income countries (LMICs)^(2)^. Inappropriate infant and young child feeding (IYCF) practices contribute to more than half of under-five children's mortality^(3)^. The first 2 years of life are recognised as a critical window in which children require more energy and nutrient-dense foods for optimal growth, physical and mental development^(4)^. Furthermore, appropriate feeding practices for infants and young children reduce morbidity, mortality and risk of other chronic diseases. Thus, ensuring adequate nutrition during the period of 6–23 months of age is a major global health priority. However, meeting nutritional needs during this age interval is challenging^(5)^.

The World Health Organization (WHO) has developed a set of core indicators to assess IYCF practices among children aged 6–23 months considering both breast- and complementary feeding-related practices^(6)^. Dietary diversity is one of the key indicators identified as a useful predictor of the nutrient adequacy of children's dietary patterns^(7)^. It is a measure of the number of different food items/groups consumed over 24 h^(6)^. The WHO identified eight food groups (breast milk, grains, roots and tubers; legumes and nuts; dairy products; flesh foods (meats/fish/poultry); eggs; vitamin A-rich fruits and vegetables; and other fruits and vegetables) which provide the required amount of macro and micronutrients for children aged 6–23 months. It is recommended that children aged 6–23 months consume at least five or more food groups daily^(8)^.

Consumption of diversified food has been linked to the better nutritional status of children in LMICs^(9,10)^. On the other hand, inadequate dietary diversity has been linked to stunting and being underweight among children. Children who do not meet the minimum dietary diversity requirements are more likely to be stunted, underweight and anaemic^(11–13)^. Furthermore, inadequately diversified diets predispose children to infection and severe illnesses^(14)^.

Globally, only 28⋅2 % of children aged 6–23 months get the recommended level of dietary diversity. The situation is exacerbated in LMICs, particularly in South Asia, Eastern, West Southern and Central Africa^(15)^. Despite efforts to improve children's dietary diversity, Ethiopia remains the country with the lowest adequate dietary diversity among East African countries^(16)^. In 2016, only 14 % of Ethiopian children had been given a sufficient number of food groups and were considered to have an adequately diverse diet^(17)^.

Few studies in Ethiopia had attempted to assess the determinants of dietary diversity among children, particularly those aged 6–23 months^(18–21)^. However, available studies are restricted to certain areas and contexts, and comprehensive evidence-based nationally representative data are scarce. Hence, we investigated the community and individual-level determinants of minimum dietary diversity among children aged 6–23 months in Ethiopia, using an advanced statistical method, and considering the hierarchical nature of the Ethiopia Demographic and Health Survey (DHS) data.

## Methods

### Study setting

The study was carried out in Ethiopia, a country located in Northeastern Africa. The country has a total estimated population of 109⋅2 million people and covers about 1⋅1 million square kilometres of area and has great geographical diversity, ranging from 4550 m above sea level to 110 m below sea level. The data were collected based on the country's previous nine administrative regions and two administrative cities^(17)^, but the country now has two additional regions (Sidama region and South West Ethiopia Region) that are separate from the Southern Nations, Nationalities and Peoples’ Region (SNNPR). The administrative region is divided into zones, districts, towns and kebeles (the smallest administrative units).

### Study design

The present study used a cross-sectional, secondary data analysis design. We used the most recent and nationally representative 2016 Ethiopian Demographic Health Survey (EDHS) data^(17)^. A stratified two-stage cluster sampling technique was applied. A total of 645 enumeration areas (EAs) were chosen in the first stage, using probabilities proportionate to EA size (202 in urban and 443 in rural) (based on the 2007 EPHC frame). A fixed number of twenty-eight households in each cluster were chosen using an equal probability systematic sampling technique in the second stage. For this study, Kids Record (KR) file containing information about women and children was used, and important variables related to inadequate dietary diversity were extracted from the dataset. In the present study, 2960 weighted data of children aged 6–23 months were used for analysis.

### Measurement of outcome

Based on the updated WHO guideline^(8)^, minimum dietary diversity was defined as the proportion of children aged 6–23 months who consumed at least five food groups out of the eight referenced food groups within 24 h. These food groups are (1) breast milk; (2) grains, roots, and tubers; (3) legumes and nuts; (4) dairy products; (5) flesh foods (meats/fish/poultry); (6) eggs; (7) vitamin A-rich fruits and vegetables; and (8) other fruits and vegetables. The total dietary diversity score ranges from 0 to 8, with 1 point given for each of the 8 food groups consumed. Children with dietary diversity scores ≥5 were classified as they attained the minimum dietary diversity, whereas those with scores <5 were classified as unmet MDD. The outcome variable was coded as 1 for adequate dietary diversity and 0 for inadequate dietary diversity.

### Determinants

We selected possible determinants based on evidence from literature and the availability of variables in the EDHS-2016. We investigated the effect of explanatory factors on dietary diversity at both the individual and community levels.

The study included individual-level determinants such as child, maternal and paternal characteristics. The children's characteristics included sex, age (in months), birth order and episodes of cough or fever in the last 2 weeks. Maternal characteristics included: age (years), highest educational level, frequency of listening to the radio, frequency of watching television, attending Antenatal care (ANC) follow-up, place of delivery, postnatal care visit and maternal empowerment^(22)^. Paternal characteristics included paternal characteristics including the highest educational level and occupation. Household characteristics include the household wealth index, the gender of the household head, the number of children under the age of five and the number of total household members. Parents’ occupations were classified as Not working (unemployed), Nonagricultural works (professional, technical, managerial, clerical, sales, services, skilled manual and unskilled manual), Agricultural works (agricultural – employee) and others.

The community-level determinants included contextual region (agrarian dominant, city dwellers dominant and pastoralist dominant), place of residence (either urban *v*. rural) and aggregate variables such as community poverty (higher *v*. lower), community distance to a health facility (distance a big problem *v*. distance, not a big problem) and remoteness of the location. Community poverties were created from mean values of wealth index categories of the individual mothers for each cluster. The two values for the community poverty level were higher poverty and lower poverty.

The EDHS did not capture data that can directly describe the characteristics of the community/clusters except the place of residence, mean rainfall, mean temperature and altitude. Hence, we created community variables by aggregating the individual-level characteristics within their clusters. The aggregates were computed using the average values of the proportions of women in each category of a given variable. Likewise, based on the national median values aggregate values were categorised into groups. These aggregate community-level determinants include contextual region, community distance to a health facility and remoteness of the location. We used GIS estimates of travel time to cities to construct a ‘living in a remote location’ dummy variable that equals 1 if the DHS cluster has more than a one-hour travel time to a town/city of 20 000 people or more^(22)^.

Contextual region: For this study, the administrative regions were categorised into agrarian, pastoralist and city, based on their settings that may have a relationship with child dietary diversity. Since regions used for administrative purposes might not necessarily be related to the child feeding practice of the population. The regions of Tigray, Amhara, Oromia, SNNPR, Gambella and Benshangul-Gumuz were recorded as agrarian. The Somali and Afar regions were combined to form the pastoralist region and the city administrations – Addis Ababa, Dire Dawa and Harar – were combined as a city. Though Gambela and Benshangul-Gumuz have been considered pastoralists in recent times, their living settings approached the agrarian^(23)^.

Ecological level variables such as mean rainfall per year, 1985–2015 (mm), mean temperature, 1985–2015 (Celsius) and altitude (metres) were also included.

### Data management and analysis

The data were analysed using STATA version 16 (StataCorp, College Station, TX, USA). In this analysis, households with children aged 6–23 months old with no missing information on dietary diversity were included. To adjust for the redistribution of samples to different regions and the possible variation in response rates, we used sampling weight in all the analyses. The ‘Svy’ command was used to allow for adjustments for the cluster sampling design. Categorical variables were reported using absolute and relative frequencies; whereas continuous variables were summarised using mean with standard deviation (sd) or median and interquartile range (IQR) for variables that deviate from normal distribution after visual examination using a histogram.

Due to the nature of the EDHS data, being a hierarchical structure, data are often correlated and thus cannot be assumed, independent. Hence, to identify individual and community-level determinants of dietary diversity, we performed a multilevel logistic regression. A multilevel approach adequately adjusts the unexplained variability of the nested structure and can estimate cluster-level effects on the outcome variable. Therefore, in the present study, a two-level mixed-effect logistic regression analysis was employed to estimate the independent (fixed) effects of the explanatory variables on dietary diversity adjusting for cluster and regional-level random effects. To investigate the community and individual-level determinants of minimum dietary diversity among children aged 6–23 months, any variable with a *P*-value of 0⋅25 on a univariable test was a candidate for the multivariable model, along with all variables of known clinical importance. Four models were fitted and compared. Model 1 was an empty model which was fitted without independent variables to test random variability using the Intraclass correlation coefficient (ICC); Model 2 include individual-level factors (age of the child, mother's educational level, frequency of listening a radio and wealth quintile of household); Model 3 include community-level factors (place of residence (either urban *v*. rural), mean annual rainfall of the cluster 1985–2015 (mm) and mean temperature 1985–2015 (Celsius)) and Model 4 include both individual and community-level factors. The relative fits of these models were then compared using the Akaike (AIC) and Bayesian Information Criterion (BIC), and the difference in model fit was compared using the *χ*^2^ test. Finally, the adjusted odds ratios (AORs) with the 95 % confidence intervals (95 % CI) were reported.

### Ethical considerations

We requested access to the datasets from the Demographic and Health Surveys (DHS) program/ICF International, and permission was granted by DHS program data archivists to download the dataset for this study. Before the authors could access the data, it was de-identified. The data were only used for the registered research topic and were not shared with anyone else.

## Results

### Participant's characteristics

Out of a total of 2960 children, the majorities (53⋅0 %) were girls, with 36⋅7 % aged 12–17 months. Nearly one-third (32 %) of children were second or third born for their families. The majority (52⋅0 %) of mothers were between the ages of 20 and 29 years, and 802 (60⋅9 %) had no formal education. More than half of the mothers (61⋅5 %) gave birth at home, and 1018 (34⋅0 %) did not seek ANC during their most recent pregnancy. The proportion of mothers who never watched television and never listened to the radio was 81⋅6 and 72⋅5 %, respectively. Two hundred and seven (7⋅4 %) of fathers and 1802 (57⋅5 %) of mothers were unemployed. Most children (87⋅8 %) resided in rural areas and agrarian dominant regions (91⋅8 %) (Table 1).

**Table 1.** Children's, parental, household, healthcare and community-level characteristics of living children aged 6–23 months, Ethiopia 2016

Characteristics	*n*	%
*Child*		
Sex		
Boy	1390	47⋅0
Girl	1570	53⋅0
Age (months)		
6–8	561	18⋅9
9–11	499	16⋅9
12–17	1085	36⋅7
18–23	816	27⋅6
Birth order		
1	576	19⋅5
2–3	934	31⋅6
4–5	626	21⋅1
6 and above	824	27⋅8
Child health: had the following symptom in the past 2 weeks		
Diarrhoea	234	7⋅9
Fever	172	5⋅8
Cough	719	24⋅3
*Maternal*		
Age (years)		
15–19	158	5⋅3
20–29	1539	52⋅0
30–39	1054	35⋅6
40–49	209	7⋅1
Highest educational level		
No education	1802	60⋅9
Primary	911	30⋅8
Secondary	161	5⋅4
Tertiary education	86	2⋅9
Frequency of listening to a radio		
Not at all	2146	72⋅5
Less than once a week	400	13⋅5
At least once a week	414	14⋅0
Frequency of watching television		
Not at all	2418	81⋅7
Less than once a week	261	8⋅8
At least once a week	281	9⋅5
ANC visit		
0	1018	34⋅4
1	120	4⋅1
2–3	781	26⋅4
4 and above visits	1042	35⋅2
Postnatal care visit		
No	2701	91⋅7
Yes	240	8⋅2
Don't know	3	0⋅1
Place of delivery		
Delivery at home	1819	61⋅5
Delivery at a health facility	1081	36⋅5
Other	60	2⋅0
Occupation		
Not working	1701	57⋅5
Nonagricultural works	594	20⋅1
Agricultural works	631	21⋅3
Others	33	1⋅1
Maternal empowerment		
Yes	2275	81⋅1
No	529	18⋅9
*Paternal*		
Highest educational level		
No education	1216	43⋅7
Primary	1167	42⋅0
Secondary	256	9⋅2
Higher	143	5⋅1
Occupation		
Not working	207	7⋅4
Nonagricultural works	687	24⋅7
Agricultural works	1794	64⋅4
Others	97	3⋅5
*Household (HH)*		
Sex of head of the household		
Male	2551	86⋅2
Female	409	13⋅8
Number of children under 5 years		
1	1128	38⋅6
2	1387	47⋅5
3 and above	407	13⋅9
Number of HH members		
<5	916	30⋅9
5–6	979	33⋅1
7–8	685	23⋅1
≥9	380	12⋅8
Wealth quantile		
Lowest	639	21⋅6
Second	627	21⋅2
Middle	637	21⋅5
Fourth	561	18⋅9
Highest	497	16⋅8
Community-level		
Residence		
Urban	361	12⋅2
Rural	2599	87⋅8
Region		
Agrarian dominant	2716	91⋅8
City dwellers	99	3⋅3
Pastoralist dominant	145	4⋅9
Remote location (high-density urban centre >1 h)		
Yes	1760	60⋅6
No	1144	39⋅4
Community distance to a health facility		
The distance is a big problem	1601	54⋅1
Distance is not a big problem	1359	45⋅9
Community poverty		
Higher poverty	1305	44⋅1
Lower poverty	1655	55⋅9
	Mean	sd
Mean rainfall per year, 2000–15 (mm)	1065⋅23	320⋅9
Mean temperature, 2000–15 (Celsius)	19⋅88	2⋅9
Altitude (metres)	1934⋅61	533⋅4

### Dietary diversity

**Fig. 1.**
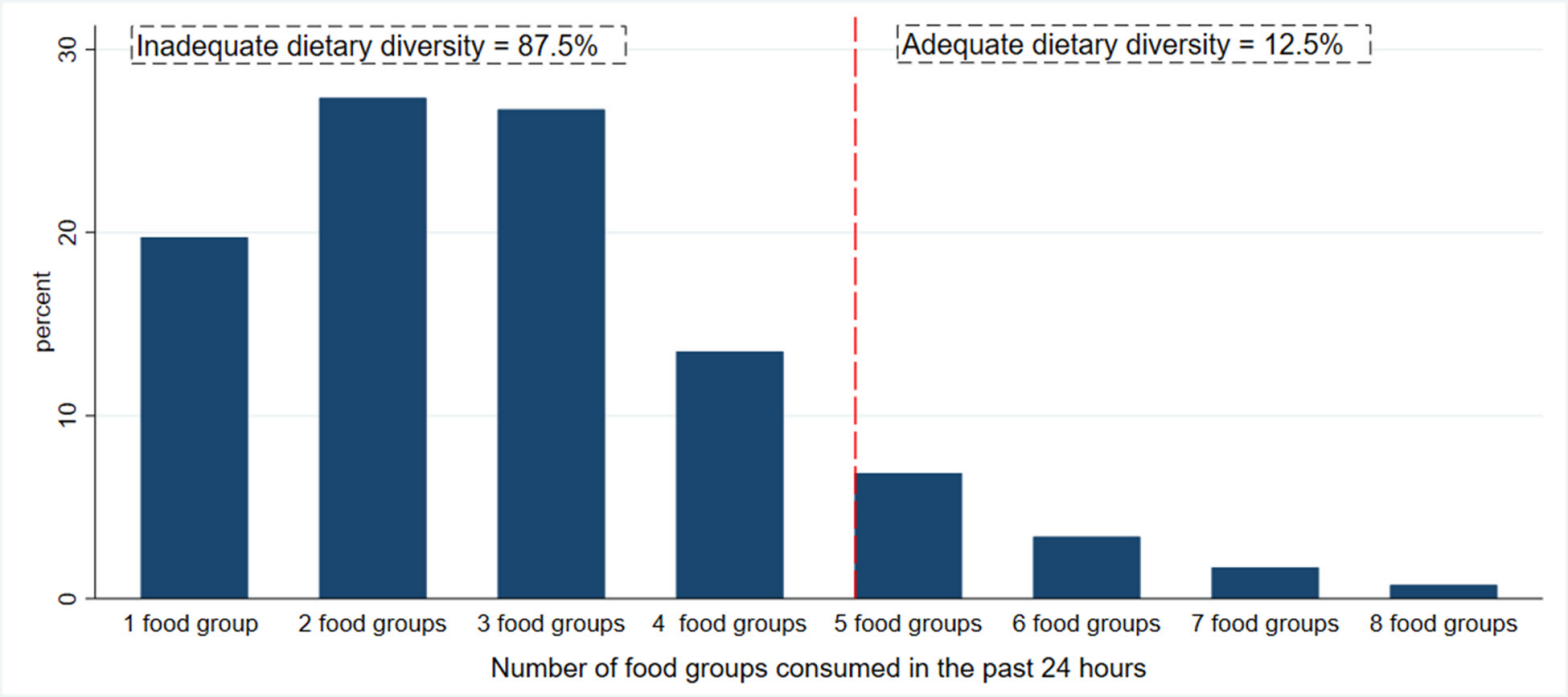
Distribution of the dietary diversity score and prevalence of the minimum dietary diversity for 6–23 months children in Ethiopia 2016.

For children aged 6–23 months, the median dietary diversity score was 4 (IQR: 2–4), with only 12⋅5 % achieving the minimum dietary diversity (Fig. 1). The proportion of children who met the required minimum dietary diversity was higher among those who reside in urban (28⋅1 %) than rural (10⋅3 %). The percentage of children who achieved the minimum dietary diversity was lower among children belonging to households in the lower quantiles (7⋅4 %) compared to second, middle, fourth and highest households. Only 9⋅7 % of children whose mother has no education has achieved the minimum dietary diversity, which is much lower compared to children whose mothers have primary (13⋅3 %), secondary (20⋅6 %) and tertiary education (46⋅5 %) education (Table 2).

**Table 2.** The distribution of children 6–23 months who achieve the minimum dietary diversity in Ethiopia 2016

Background characteristic	*N*	%
Age in months		
6–8 months	27	4⋅8
9–11 months	69	13⋅8
12–17 months	184	17⋅0
18–23 months	90	11⋅0
Sex of the child		
Boy	182	13⋅1
Girl	188	12⋅0
Residence		
Urban	102	28⋅1
Rural	268	10⋅3
Mother's highest education level		
No education	175	9⋅7
Primary	122	13⋅3
Secondary	33	20⋅6
Higher	40	46⋅5
Wealth quantile		
Lowest	47	7⋅4
Second	69	11⋅0
Middle	68	11⋅6
Fourth	89	16⋅0
Highest	96	19⋅4

### Food groups consumed

Grain, roots and tubers (63⋅6 %) were the most consumed food items 24 h preceding the survey. However, only 8⋅7 and 17⋅2 % of children, respectively, have consumed flesh foods and eggs in the past 24 h preceding the survey. In addition, only 2⋅8 % of children aged 6–8 months consumed flesh foods such as meat and poultry (Table 3).

**Table 3.** The distribution of intake of eight food groups for children 6–23 months of age, in Ethiopia 2016

Food groups	All subjects	6–8 months	9–11 months	12–17 months	18–23 months
	*n* (%)	*n* (%)	*n* (%)	*n* (%)	*n* (%)
Grain, roots and tubers	1883 (63⋅6)	216 (39⋅2)	307 (61⋅4)	744 (68⋅5)	613 (75⋅2)
Legumes and nuts	633 (21⋅4)	57 (10⋅1)	133 (26⋅7)	239 (22⋅0)	204 (25⋅00
Dairy products	1152 (38⋅9)	199 (35⋅6)	206 (41⋅2)	416 (38⋅4)	330 (40⋅5)
Flesh foods	258 (8⋅7)	16 (2⋅8)	31 (6⋅3)	124 (11⋅5)	86 (10⋅6)
Eggs	510 (17⋅2)	52 (9⋅3)	98 (19⋅6)	200 (18⋅4)	160 (19⋅6)
Vitamin A-rich fruits and vegetables	836 (28⋅2)	79 (14⋅0)	132 (26⋅4)	353 (32⋅5)	273 (33⋅5)
Other fruits and vegetables	305 (10⋅3)	28 (4⋅9)	57 (11⋅5)	117 (10⋅8)	103 (12⋅6)
Breast milk	2607 (88⋅1)	533 (95⋅1)	461 (92⋅8)	991 (91⋅4)	620 (76⋅0)

### Determinants of minimum dietary diversity

Compared to those mothers who had no exposure to listening to a radio, those who had listened to the radio at least once a week (AOR 1⋅6; 95 % CI 1⋅1, 2⋅4) or less than once a week (AOR 1⋅6; 95 % CI 1⋅1, 2⋅4) are nearly twice more likely to meet the minimum dietary diversity. Furthermore, compared to the children from the lowest wealth quintile household, those who were from the second wealth quintile household were nearly two times more likely (AOR 1⋅8; 95 % CI 1⋅1, 3⋅0), from the fourth wealth quintile household were nearly three times more likely (AOR 2⋅8; 95 % CI 1⋅6, 4⋅9) and from the highest wealth quintile household were two times more likely (AOR 2⋅1; 95 % CI 1⋅1, 4⋅0) to meet the minimum dietary diversity (Table 4).

**Table 4.** Determinants of minimum dietary diversity in children aged 6–23 months in Ethiopia 2016

Characteristic			Model 2 Individual characteristics		Model 3 Community characteristics		Model 4 Individual & community characteristics	
	COR (95 % CI)	*P*-value	AOR (95 % CI)	*P*-value	AOR (95 % CI)	*P*-value	AOR (95 % CI)	*P*-value
Age in months								
6–8 months	1⋅0 (referent)		1⋅0 (referent)				1⋅0 (referent)	
9–11 months	3⋅1 (1⋅9, 5⋅0)	<0⋅001	3⋅3 (1⋅9, 5⋅7)	<0⋅001			3⋅2 (1⋅8, 5⋅6)	<0⋅001
12–17 months	3⋅2 (2⋅1,4⋅9)	<0⋅001	4⋅0 (2⋅4, 6⋅6)	<0⋅001			4⋅0 (2⋅4, 6⋅7)	<0⋅001
18–23 months	3⋅0 (1⋅9, 4⋅7)	<0⋅001	3⋅5 (2⋅1, 5⋅9)	<0⋅001			3⋅4 (2⋅0, 5⋅8)	<0⋅001
Mother's highest educational level								
No education	1⋅0 (referent)		1⋅0 (referent)				1⋅0 (referent)	
Primary	1⋅9 (1⋅4, 2⋅5)	<0⋅001	1⋅2 (0⋅8, 1⋅7)	0⋅300			1⋅1 (0⋅8, 1⋅6)	0⋅450
Secondary	3⋅1 (2⋅2, 4⋅5)	<0⋅001	1⋅2 (1⋅0, 2⋅7)	0⋅070			1⋅2 (0⋅7, 2⋅1)	0⋅490
Tertiary education	7⋅5 (5⋅0, 11⋅1)	<0⋅001	2⋅6 (1⋅4, 5⋅2)	<0⋅001			1⋅9 (1⋅0, 3⋅7)	0⋅050
ANC visit								
0			1⋅00 (referent)				1⋅0 (referent)	
1	1⋅7 (1⋅0, 3⋅1)	0⋅063	2⋅1 (1⋅0, 4⋅3)				1⋅8 (0⋅7, 1⋅7)	0⋅100
2–3	1⋅7 (1⋅2, 2⋅3)	0⋅005	1⋅4 (0⋅9, 2⋅1)				1⋅2 (0⋅8, 3⋅7)	0⋅400
4 and above visits	2⋅6 (1⋅9, 3⋅5)	<0⋅001	1⋅4 (0⋅9, 2⋅1)				1⋅1 (0⋅7, 1⋅7)	0⋅750
Frequency of listening to a radio								
Not at all	1⋅0 (referent)		1⋅0 (referent)				1⋅0 (referent)	
Less than once a week	2⋅3 (1⋅6, 3⋅3)	<0⋅001	1⋅8 (1⋅2, 2⋅7)	<0⋅001			1⋅6 (1⋅1, 2⋅4)	0⋅020
At least once a week	4⋅0 (3⋅0, 5⋅2)	<0⋅001	1⋅7 (1⋅1, 2⋅5)	0⋅010			1⋅6 (1⋅1, 2⋅4)	0⋅020
Residence								
Urban	1⋅0 (referent)							
Rural	0⋅3 (0⋅2, 0⋅3)	<0⋅001			0⋅3 (0⋅2, 0⋅4)	<0⋅001	0⋅4 (0⋅2, 0⋅6)	<0⋅001
Wealth quantile								
Lowest	1⋅0 (referent)		1⋅0 (referent)				1⋅0 (referent)	
Second	2⋅6 (1⋅7, 3⋅9)	<0⋅001	2⋅1 (1⋅3, 3⋅6)	<0⋅001			1⋅8 (1⋅1, 3⋅1)	0⋅030
Middle	3⋅2 (2⋅1, 4⋅7)	<0⋅001	2⋅3 (1⋅3, 3⋅9	<0⋅001			1⋅6 (0⋅9, 2⋅8)	0⋅080
Fourth	5⋅4 (3⋅6, 8⋅0)	<0⋅001	3⋅6 (2⋅1, 6⋅2)	<0⋅001			2⋅9 (1⋅6, 5⋅2)	<0⋅001
Highest	6⋅2 (4⋅2, 9⋅2)	<0⋅001	2⋅7 (1⋅5, 4⋅8)	<0⋅001			2⋅2 (1⋅1, 4⋅2)	0⋅020
Mean rainfall per year, 2000–15 (mm)	1⋅0 (1⋅0, 1⋅0)	<0⋅001			1⋅0 (1⋅0, 1⋅0)	0⋅030	1⋅0 (1⋅0, 1⋅0)	0⋅100
Mean temperature, 2000–15 (Celsius)	0⋅9 (0⋅9, 1⋅0)	<0⋅001			1⋅1 (1⋅0, 1⋅1)	0⋅68	1⋅0 (1⋅0, 1⋅1)	0⋅380

COR, crude odds ratio; AOR, adjusted odds ratio; CI, confidence interval.

## Discussion

The present study aimed to assess the determinants of dietary diversity using nationally representative 2016 EDHS data. We found that only 12⋅5 % of children aged 6–23 months have adequate minimum dietary diversity, indicating a suboptimal level of dietary diversity score. In addition, we observed that only a small proportion of children consumed animal-source foods. Furthermore, the child's age, caregivers’ frequency of radio listening, residence and household wealth index were all significantly associated with children's minimum dietary diversity.

The prevalence of minimum dietary diversity was found to be lower in this study than in the 2016 EDHS report^(17)^. This could be due to the disparity in cut-off points and the number of food groups used to calculate the dietary diversity score. The previous dietary diversity score was constructed based on seven food groups and a child consuming at least four food groups is considered to have achieved the minimum dietary diversity; however, according to the most recent WHO guidelines, the dietary diversity is based on eight food groups, and a child consuming at least five of the food groups is considered to have achieved the minimum dietary diversity^(6,8)^. Furthermore, the low level of dietary diversity in the study indicates that the majority of children consume a monotonous diet that focuses on a limited number of food groups. Similarly, a systematic review of Ethiopian studies found a low suboptimal level of dietary diversity (23⋅25 %) among children aged 6–23 months^(24)^. According to studies conducted in India, Rwanda and Burundi, only a small proportion of children receive the required minimum dietary diversity^(25,26)^. This low dietary diversity may indicate an insufficient intake of micronutrients^(27)^. Therefore, National Nutrition Program in collaboration with other sectors needs to put an effort to improve the accessibility and utilisation of diversified food types to improve the dietary diversity of children.

Our study showed that grains, roots and tubers were the most consumed food items. Another study conducted in southern Ethiopia found that grains, roots and tubers are the primary staples of children aged 6–23 months^(28)^. Meanwhile, animal-source foods such as meat, dairy products and eggs are consumed only by a small proportion of children in the present study, which is consistent with studies conducted in other parts of Ethiopia^(29,30–32)^. Similarly, animal-source food consumption is lower in sub-Saharan African countries than in other parts of the world^(22)^. Animal-source foods contain a variety of vitamins and minerals, including calcium, iron, vitamin B-12, vitamin A, riboflavin and zinc, which are difficult to obtain in sufficient quantities from plant-based foods alone^(33)^. Hence, inadequate intake of animal-source foods may lead to inadequate intake of these micronutrients^(34)^. One reason for the low consumption could be that most animal-source foods were intended for market and a profitable income rather than consumption by family members. Another reason could be a lack of knowledge about the nutritional benefits of these foods^(35)^. A study conducted on Ethiopian mothers showed that mothers are afraid of feeding their children animal-source foods because they believe their children are incapable of digesting them^(36)^. As a result, we recommend that public health practitioners should increase parental and caregiver awareness of the importance of animal-source foods for child growth and development.

Additionally, low consumption of animal-source foods may be due to a lack of access caused by economic constraints^(37)^. The high price of animal-source foods does not encourage consumption by poor households. This can further be explained by the observed association between household economic status – as measured by wealth quantile and inadequate dietary diversity in this study. Children from the highest wealth quintile of households were more likely to achieve the minimum dietary diversity than children from the lowest wealth quintile of households. This finding is in line with several studies conducted in different parts of Ethiopia and other parts of the world^(32,38–40)^. This association could be explained by the fact that second, middle, fourth and highest households are more likely to be food secure and can purchase a variety of foods^(41)^.

In our study, older children had a higher odds of achieving minimum dietary diversity than younger children, indicating that infants receive inadequately diversified food when compared to older children. This might be due to the delayed introduction of complementary foods or to the use of only milk and cereals as complementary foods. Another possible explanation is that mothers believe that certain foods, such as bananas, eggs, pumpkin, carrots, green vegetables and meat, are difficult for young children to digest^(42)^. This finding is supported by similar studies conducted in Ethiopia among children aged 6–23 months^(32,40,43,44)^.

The present study showed that mothers who listen to the radio at least once a week are more likely to feed their children a diverse diet than those who do not. Consistently, a study done in India found that listening to the radio was a significant factor in children's dietary diversity^(44)^. Similar studies conducted in Northwest Ethiopia and Bangladesh found that a mother's exposure to mass media was a significant predictor of her children's dietary diversity^(32,45)^. This suggests that disseminating information about IYCF through the media may have a positive impact on their children's dietary diversity^(46)^.

Even though studies conducted in Ethiopia and Asia have found a positive association between maternal education and children's dietary diversity^(43,47–49)^, we did not find a statistically significant association between mother's educational status and children's minimum dietary diversity. This suggests that, regardless of the mother's educational attainment, there may be difficulties in translating education into improved dietary practices and subsequent improvements in nutritional status^(49)^. The other reason might be due to the presence of a very low number of mothers with the highest educational level.

The study's findings should be interpreted considering the following strengths and limitations. First, the use of nationally representative survey data from Demographic Health Surveys enhances the generalizability of our findings. Second, the application of multilevel analysis to account for individual and community-level contributors to children's dietary diversity. In Ethiopia due to religious or societal reasons, animal-source foods are commonly consumed during feast days, holidays and on special occasions or avoided during the fasting period^(35)^. We do not know whether the data were collected on a representative day, and the data collection period could have resulted in misclassification bias. The data collection period coincided with the longest religious fasting period, which may have resulted in an underestimation of animal-source food consumption, and as a result, a low dietary diversity score.

In conclusion, a suboptimal level of dietary diversity has been observed among Ethiopian children aged 6–23 months. This low dietary diversity is more prevalent in rural children and children from low-income families. The present study also found that individual and community-level factors such as a child's age, frequency of radio listening, residence and household wealth status influence children's dietary diversity. Nutrition programmes promoting dietary diversity should be implemented throughout the country, particularly for those from low-income families living in rural areas, in collaboration with health workers and/or the media. Furthermore, reinforcing optimal feeding practices through nutrition education provided by the healthcare providers will assist parents and other caregivers in providing a more diverse diet for their children. Further studies should assess community and individual-level determinants of adequate minimum dietary diversity using representative data.

## References

[1] Black RE, Victora CG, Walker SP, (2013) Maternal and child undernutrition and overweight in low-income and middle-income countries. Lancet 382, 427–451. doi:10.1016/S0140-6736(13)60937-X, Epub 2013 Jun 6. Erratum in: *Lancet* 2013;**382**(9890):396. PMID: 23746772. 23746772

[2] WHO (2020) Malnutrition. https://www.who.int/news-room/fact-sheets/detail/malnutrition (accessed 5 January 2021).

[3] Nutrition IC (2013) The Achievable Imperative for Global Progress. New York, NY: UNICEF.

[4] World Health Organization (2009) Guiding Principles for Feeding Non-breastfed Children 6–24 Months of Age. 2005. Geneva: WHO.

[5] Dewey KG (2013) The challenge of meeting nutrient needs of infants and young children during the period of complementary feeding: an evolutionary perspective. J Nutr 143, 2050–2054. doi:10.3945/jn.113.182527, Epub 2013 Oct 16. PMID: 24132575; PMCID: PMC3827643. 24132575 PMC3827643

[6] World Health Organization (2010) Indicators for Assessing Infant and Young Child Feeding Practices Part 3: Country Profiles. Geneva, Switzerland: World Health Organization.

[7] Nti CA (2011) Dietary diversity is associated with nutrient intakes and nutritional status of children in Ghana. Asian J Med Sci 2, 105–109.

[8] World Health Organization (2017) Global Nutrition Monitoring Framework: operational guidance for tracking progress. Geneva, Switzerland: World Health Organization.

[9] Arimond M & Ruel MT (2004) Dietary diversity is associated with child nutritional status: evidence from 11 Demographic and Health Surveys. J Nutr 134, 2579–2585. doi:10.1093/jn/134.10.2579, PMID: 15465751. 15465751

[10] Sawadogo PS, Martin-Prével Y, Savy M, (2006) An infant and child feeding index is associated with the nutritional status of 6- to 23-month-old children in rural Burkina Faso. J Nutr 136, 656–663. doi:10.1093/jn/136.3.656, PMID: 16484539. 16484539

[11] Rah JH, Akhter N, Semba RD, (2010) Low dietary diversity is a predictor of child stunting in rural Bangladesh. Eur J Clin Nutr 64, 1393–1398. doi:10.1038/ejcn.2010.171, Epub 2010 Sep 15. PMID: 20842167. 20842167

[12] Khamis AG, Mwanri AW, Ntwenya JE, (2019) The influence of dietary diversity on the nutritional status of children between 6 and 23 months of age in Tanzania. BMC Pediatr 19, 518. doi:10.1186/s12887-019-1897-5, PMID: 31881999; PMCID: PMC6935228. 31881999 PMC6935228

[13] Belachew A & Tewabe T (2020) Under-five anemia and its associated factors with dietary diversity, food security, stunted, and deworming in Ethiopia: systematic review and meta-analysis. Syst Rev 9, 31. doi:10.1186/s13643-020-01289-7, PMID: 32051034; PMCID: PMC7017616. 32051034 PMC7017616

[14] Caulfield LE, de Onis M, Blössner M, (2004) Undernutrition as an underlying cause of child deaths associated with diarrhea, pneumonia, malaria, and measles. Am J Clin Nutr 80, 193–198. doi:10.1093/ajcn/80.1.193, PMID: 15213048. 15213048

[15] White JM, Bégin F, Kumapley R, (2017) Complementary feeding practices: current global and regional estimates. Matern Child Nutr 13, e12505. doi:10.1111/mcn.12505, PMID: 29032623; PMCID: PMC6865887. PMC6865887 29032623

[16] Kang Y, Chimanya K, Matji J, (2019) Determinants of minimum dietary diversity among children aged 6–23 months in 7 countries in East and Southern Africa (P10-035-19). Curr Dev Nutr 3, nzz034-P10.

[17] Ethiopian Public Health Institute (EPHI), ICF (2017) Ethiopia Demographic and Health Survey 2016: Final Report. Rockville, Maryland, USA.

[18] Kuche D, Moss C, Eshetu S, (2020) Factors associated with dietary diversity and length-for-age z-score in rural Ethiopian children aged 6–23 months: a novel approach to the analysis of baseline data from the Sustainable Undernutrition Reduction in Ethiopia evaluation. Matern Child Nutr 16, e12852. doi:10.1111/mcn.12852, Epub 2019 Jul 13. PMID: 31124274; PMCID: PMC7038872. 31124274 PMC7038872

[19] Gatahun EA & Abyu DM (2015) Dietary diversity feeding practice and determinants among children aged 6–23 months in Kemba Woreda, southern Ethiopia implication for public health intervention. J Nutr Food Sci 13, S13003.

[20] Dangura D & Gebremedhin S (2017) Dietary diversity and associated factors among children 6-23 months of age in Gorche district, Southern Ethiopia: cross-sectional study. BMC Pediatr 17, 6. doi:10.1186/s12887-016-0764-x, PMID: 28068965; PMCID: PMC5223415. 28068965 PMC5223415

[21] Disha A, Tharaney M, Abebe Y, (2015) Factors Associated with Infant and Young Child Feeding Practices in Amhara Region and Nationally in Ethiopia: Analysis of the 2005 and 2011 Demographic and Health Surveys, vol. 2, pp. 6–59, Washington, DC: Alive and Thrive.

[22] Choudhury S, Headey DD & Masters WA (2019) First foods: diet quality among infants aged 6–23 months in 42 countries. Food Policy 88, 101762. doi:10.1016/j.foodpol.2019.101762, PMID: 31853163; PMCID: PMC6894322. 31853163 PMC6894322

[23] Yebyo H, Alemayehu M & Kahsay A (2015) Why do women deliver at home? Multilevel modeling of Ethiopian National Demographic and Health Survey data. PLoS One 10, e0124718. doi:10.1371/journal.pone.0124718, PMID: 25874886; PMCID: PMC4398378. 25874886 PMC4398378

[24] Temesgen H, Negesse A, Woyraw W, (2018) Dietary diversity feeding practice and its associated factors among children age 6-23 months in Ethiopia from 2011 up to 2018: a systematic review and meta-analysis. Ital J Pediatr 44, 109. doi:10.1186/s13052-018-0567-9, PMID: 30223854; PMCID: PMC6142683. 30223854 PMC6142683

[25] Beckerman-Hsu JP, Kim R, Sharma S, (2020) Dietary variation among children meeting and not meeting minimum dietary diversity: an empirical investigation of food group consumption patterns among 73,036 children in India. J Nutr 150, 2818–2824. doi:10.1093/jn/nxaa223, PMID: 32805040; PMCID: PMC7762760. 32805040 PMC7762760

[26] Custodio E, Herrador Z, Nkunzimana T, (2019) Children's dietary diversity and related factors in Rwanda and Burundi: a multilevel analysis using 2010 Demographic and Health Surveys. PLoS One 14, e0223237. doi:10.1371/journal.pone.0223237, PMID: 31596868; PMCID: PMC6785172. 31596868 PMC6785172

[27] Kennedy GL, Pedro MR, Seghieri C, (2007) Dietary diversity score is a useful indicator of micronutrient intake in non-breast-feeding Filipino children. J Nutr 137, 472–477. doi:10.1093/jn/137.2.472, PMID: 17237329. 17237329

[28] Temesgen M (2013) Nutritional status of Ethiopian weaning and complementary foods: a review. Open Access Sci Rep 2, 1–9.

[29] Feyisa BB, Tefera GM, Endris BS, (2020) Feeding practice, energy, and nutrient intake adequacy among children aged 6–23 months in southern Ethiopia: a community based cross-sectional study. Food Sci Nutr 8, 6680–6690. doi:10.1002/fsn3.1962, PMID: 33312551; PMCID: PMC7723221. 33312551 PMC7723221

[30] Tessema M, Belachew T & Ersino G (2013) Feeding patterns and stunting during early childhood in rural communities of Sidama, South Ethiopia. Pan Afr Med J 14, 75. doi:10.11604/pamj.2013.14.75.1630, PMID: 23646211; PMCID: PMC3641921. 23646211 PMC3641921

[31] Temesgen H, Yeneabat T & Teshome M (2018) Dietary diversity and associated factors among children aged 6–23 months in Sinan Woreda, Northwest Ethiopia: a cross-sectional study. BMC Nutr 4, 5. doi:10.1186/s40795-018-0214-2, PMID: 32153869; PMCID: PMC7050891. 32153869 PMC7050891

[32] Beyene M, Worku AG & Wassie MM (2015) Dietary diversity, meal frequency and associated factors among infant and young children in Northwest Ethiopia: a cross-sectional study. BMC Public Health 15, 1007. doi:10.1186/s12889-015-2333-x, PMID: 26433689; PMCID: PMC4592571. 26433689 PMC4592571

[33] Neumann C, Harris DM & Rogers LM (2002) Contribution of animal source foods in improving diet quality and function in children in the developing world. Nutr Res 22, 193–220.

[34] Dror DK & Allen LH (2011) The importance of milk and other animal-source foods for children in low-income countries. Food Nutr Bull 32, 227–243. doi:10.1177/156482651103200307, PMID: 22073797. 22073797

[35] Haileselassie M, Redae G, Berhe G, (2020) Why are animal source foods rarely consumed by 6–23 months old children in rural communities of Northern Ethiopia? A qualitative study. PLoS One 15, e0225707. doi:10.1371/journal.pone.0225707. Erratum in: *PLoS One* 2020;**15**(3):e0230527. PMID: 31914130; PMCID: PMC6948827. 31914130 PMC6948827

[36] Alive & Thrive (2010) IYCF practices, beliefs and influences in Tigray region, Ethiopia. Addis Ababa, Ethiopia: Alive & Thrive.

[37] Gebremedhin S, Baye K, Bekele T, (2017) Predictors of dietary diversity in children ages 6 to 23 mo in largely food-insecure area of South Wollo, Ethiopia. Nutrition 33, 163–168. doi:10.1016/j.nut.2016.06.002, Epub 2016 Jun 16. PMID: 27499206. 27499206

[38] Joshi N, Agho KE, Dibley MJ, (2012) Determinants of inappropriate complementary feeding practices in young children in Nepal: secondary data analysis of Demographic and Health Survey 2006. Matern Child Nutr 8, 45–59. doi:10.1111/j.1740-8709.2011.00384.x, PMID: 22168518; PMCID: PMC6860874. 22168518 PMC6860874

[39] Solomon D, Aderaw Z & Tegegne TK (2017) Minimum dietary diversity and associated factors among children aged 6-23 months in Addis Ababa, Ethiopia. Int J Equity Health 16, 181. doi:10.1186/s12939-017-0680-1, PMID: 29025434; PMCID: PMC5639776. 29025434 PMC5639776

[40] Belew AK, Ali BM, Abebe Z, (2017) Dietary diversity and meal frequency among infant and young children: a community based study. Ital J Pediatr 43, 73. doi:10.1186/s13052-017-0384-6, PMID: 28810887; PMCID: PMC5558775. 28810887 PMC5558775

[41] Dafursa K & Gebremedhin S (2019) Dietary diversity among children aged 6–23 months in Aleta Wondo District, Southern Ethiopia. J Nutr Metab 2019, 2869424. doi:10.1155/2019/2869424, PMID: 31815015; PMCID: PMC6878804. 31815015 PMC6878804

[42] Tassew AA, Tekle DY, Belachew AB, (2019) Factors affecting feeding 6-23 months age children according to minimum acceptable diet in Ethiopia: a multilevel analysis of the Ethiopian Demographic Health Survey. PLoS One 14, e0203098. doi:10.1371/journal.pone.0203098, PMID: 30789922; PMCID: PMC6383941. 30789922 PMC6383941

[43] Tegegne M, Sileshi S, Benti T, (2017) Factors associated with minimal meal frequency and dietary diversity practices among infants and young children in the predominantly agrarian society of Bale zone, Southeast Ethiopia: a community based cross sectional study. Arch Public Health 75, 53. doi:10.1186/s13690-017-0216-6, PMID: 29158896; PMCID: PMC5682638. 29158896 PMC5682638

[44] Patel A, Pusdekar Y, Badhoniya N, (2012) Determinants of inappropriate complementary feeding practices in young children in India: secondary analysis of National Family Health Survey 2005–2006. Matern Child Nutr 8, 28–44. doi:10.1111/j.1740-8709.2011.00385.x, PMID: 22168517; PMCID: PMC6860525. 22168517 PMC6860525

[45] Blackstone S & Sanghvi T (2018) A comparison of minimum dietary diversity in Bangladesh in 2011 and 2014. Matern Child Nutr 14, e12609. doi:10.1111/mcn.12609, Epub 2018 Apr 16. PMID: 29663657; PMCID: PMC6866105. 29663657 PMC6866105

[46] Bedada Damtie S, Benti Tefera T & Tegegne Haile M (2020) Dietary diversity practice and associated factors among children aged 6–23 months in Robe Town, Bale zone, Ethiopia. J Nutr Metab 2020, 9190458. doi:10.1155/2020/9190458, PMID: 32685209; PMCID: PMC7350076. 32685209 PMC7350076

[47] Gautam KP, Adhikari M, Khatri RB, (2016) Determinants of infant and young child feeding practices in Rupandehi, Nepal. BMC Res Notes 9, 135. doi:10.1186/s13104-016-1956-z, PMID: 26936368; PMCID: PMC4776375. 26936368 PMC4776375

[48] Kabir I, Khanam M, Agho KE, (2012) Determinants of inappropriate complementary feeding practices in infant and young children in Bangladesh: secondary data analysis of Demographic Health Survey 2007. Matern Child Nutr 8, 11–27. doi:10.1111/j.1740-8709.2011.00379.x, PMID: 22168516; PMCID: PMC6860519. 22168516 PMC6860519

[49] Khan AM, Kayina P, Agrawal P, (2012) A study on infant and young child feeding practices among mothers attending an urban health center in East Delhi. Indian J Public Health 56, 301–304. doi:10.4103/0019-557X.106420, PMID: 23354143. 23354143

